# Nutritional Value, Antinutritional Factors, and Protein Quality of *Brosimum alicastrum* Seeds: A Sustainable Protein Source

**DOI:** 10.1021/acs.jafc.5c06101

**Published:** 2025-09-05

**Authors:** Hani Farhana Nazir, Raul Tapia-Tussell, Matthew G. Nosworthy, Oscar Abel Sánchez-Velázquez, Adam Dowle, Idolo Ifie, Alan Javier Hernández-Álvarez

**Affiliations:** † School of Food Science & Nutrition, 4468 University of Leeds, Leeds LS2 9JT, U.K.; ‡ Renewable Energy Unit, Yucatan Scientific Research Center, Sierra Papacal, Merida 97302, Mexico; § Guelph Research and Development Centre, Agriculture and Agri-Food Canada, Guelph, Ontario N1G 5C9, Canada; ∥ College of Pharmacy and Nutrition, University of Saskatchewan, Saskatoon, Saskatchewan S7N 5E5, Canada; ⊥ Bioscience Technology Facility, Department of Biology, 8748 University of York, York YO10 5DD, U.K.; # National Alternative Protein Innovation Centre, NAPIC, Leeds LS2 9JT, U.K.

**Keywords:** Ramon tree, *Brosimum alicastrum*, antinutritional factors, amino acid profile, protein digestibility, protein quality

## Abstract

This study evaluated the nutritional and antinutritional (ANFs) composition and protein profiles of different components of Ramon (*Brosimum alicastrum*) seed, including the seed coat, fruit, and both roasted and green (unprocessed) seeds. Proximate composition, mineral content, ANFs quantification, amino acid profile, *in vitro* protein digestibility, SDS-PAGE, proteomics, and gluten ELISA were performed. Protein contents ranged from 9.85 to 10.69 g/100 g. ANFs—saponins (961.10–1337.58 mg DE/100 g), tannins (12.67–208.66 mg CE/100 g), phytic acid (1327.88–3592.51 mg/100 g), and oxalates (365.08–1431.48 mg CaC_2_O_4_/100 g)—varied by processing. *In vitro* digestibility-corrected amino acid scores (25.05–47.85%) confirmed low to moderate digestibility. SDS-PAGE showed low-molecular-weight proteins (<25 kDa) predominantly, and mass spectrometry corroborated the presence of β-amylase and glucan-phosphorylase proteins. Gluten ELISA analysis confirmed Ramon flour is gluten-free. These results highlight Ramon seeds as a sustainable, nutrient-dense, gluten-free protein source suitable for functional food applications, addressing future protein security needs.

## Graphical abstract

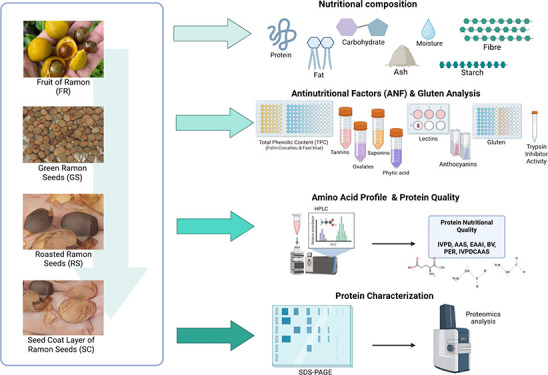

## 1 Introduction

Given projected global population growth nearing 9.7 billion by 2050, ensuring equitable access to nutritionally adequate and safe food is a critical contemporary challenge.^1^ In response to increasing consumer interest in environmentally sustainable alternative protein sources that meet human nutritional requirements, the seed of the Ramon tree (Moraceae: *Brosimum alicastrum*) has gained attention.^2,3^ This tree, which has a long history of use within Mayan culture, is indigenous to the tropical forests of Mexico and Central America.^4^

Ethnobotanical studies documenting the medicinal use of Ramon seeds in Yucatan and Jalisco, Mexico, date back to the first half of the 20th century.^5,6^ While these seeds have been a seasonal food source for local communities, their nutritional applications have only recently garnered significant attention. Quintero-Hilario et al.^3^ demonstrated that Ramon seeds are a rich source of dietary fiber, minerals, folic acid, vitamins, and essential amino acids (EAA). Furthermore, Subiria-Cueto et al.^7^ reported that Ramon seed flour contains 13.0 g/100 g dietary fiber (fresh weight basis, fwb), a substantially higher value than that of commercial wheat flour (1.6 g/100 g fwb). Carter^2^ determined that the protein content of Ramon seeds varies depending on processing, ranging from 8.80–12.13% in dried seeds, 10.42–11.48% in roasted seeds, and 11.48% in cooked seeds, highlighting the potential of these seeds for the production of protein ingredients with enhanced functionalities.

Ramon seeds contain substantial quantities of essential minerals, including potassium (K, 1180 mg/100 g), calcium (Ca, 98 mg/100 g), iron (Fe, 2.09 mg/100 g), and magnesium (Mg, 68 mg/100 g).^8^ Fresh green seeds exhibit higher concentrations of potassium and calcium compared to roasted seeds.^2,9,10^ Notably, the potassium and calcium levels in Ramon seeds surpass those observed in various nuts such as pecan, hazelnut, macadamia, and pistachio (363–1007 mg/100 g K and 54–123 mg/100 g Ca).^11^

Despite the promising nutritional profile of Ramon seeds, it is crucial to recognize that, as with many plant-based foods, they might contain antinutritional factors (ANFs) such as saponins, tannins, phytic acid, phenolics, anthocyanins, and trypsin inhibitors, which can potentially diminish nutrient digestibility and absorption in humans.^12^ This potential for reduced bioavailability necessitates further investigation, as currently, no published studies have specifically explored the presence of these compounds in Ramon seeds.

Beyond the general presence of ANFs, protein quality is a critical parameter for evaluating the potential nutritional benefits of novel food sources. Protein digestibility is a critical metric for evaluating food proteins, as it directly determines protein bioaccessibility, the release of bioactive peptides, and the availability of essential and nonessential amino acids. Protein digestibility is inherently affected by the presence of ANFs and plays a pivotal role in determining the overall nutritional quality of plant proteins.^13^ Furthermore, the extent of protein digestibility can significantly impact other functional attributes, including the bioactivity and nutritional performance of Ramon seed proteins. These considerations underscore the need for comprehensive studies to fully assess their potential contribution to human nutrition.

The aims of this research were (1) to determine the nutritional composition, (2) to analyze a broad spectrum of ANFs (including anthocyanins, oxalates, phytic acid, saponins, trypsin inhibitors, phenolics, tannins, and lectins) of different components of Ramon seed including the seed coat, fruit, and both roasted and green (unprocessed) seeds, and (3) to assess the protein quality through *in vitro* protein digestibility (IVPD), amino acid composition, and protein quality indices including amino acid score (AAS), essential amino acid index (EAAI), biological value (BV), protein efficiency ratio (PER), and *in vitro* protein digestibility corrected amino acid score (IVPDCAAS). Furthermore, a proteomics analysis was performed to identify the specific proteins present in the*Brosimum alicastrum* components of the Ramon seed. To the best of our knowledge, this study is the first to provide a comprehensive overview and comparative assessment of the nutritional composition, a broad range of ANFs, protein quality parameters, and protein identification of different components of Ramon seed. Through this research, we aim to provide a broader understanding of the applicability, protein quality, and overall composition of Ramon seeds.

## 2 Materials and Methods

### 2.1 Ramon Seeds Procurement and Reagents

The mature fruits of Ramon (*Brosimum alicastrum*) were provided by Centro de Investigación Cientfica de Yucatán (CICY) from Ramon trees located at San Antonio Hool, Yucatán, Mexico (21°3′54″ N, 89°40′30″ W; 7 m a.s.l.). This study assessed different components of Ramon, including the fruit (FR), green (unprocessed) seeds (GS), roasted seeds (RS), and seed coat layer (SC) (Figure S1).

All chemicals and reagents used in this study were of analytical grade. Methanol, acetone, formic acid, hydrochloric acid (HCl), sodium carbonate, ethylenediaminetetraacetic acid (EDTA), vanillin, sulfuric acid (H_2_SO_4_), calcium chloride (CaCl_2_), potassium permanganate (KMnO_4_), ammonium hydroxide (NH_4_OH), sodium chloride (NaCl), sulfosalicylic acid, ferric chloride (FeCl_3_·6H_2_O), and all amino acid standards were purchased from Sigma-Aldrich (St. Louis, MO, USA) unless otherwise stated. Diosgenin and catechin standards were also obtained from Sigma-Aldrich. The Folin–Ciocalteu reagent was sourced from Merck (Darmstadt, Germany). All buffer components, including phosphate-buffered saline (PBS), were prepared with Milli-Q water (Merck Millipore, Burlington, MA, USA). The Wheat/Gluten (Gliadin) ELISA Kit was purchased from Crystal Chem (Elk Grove, IL, USA). The Sheep Hemagglutination Kit (KPA-3913) was obtained from Klinipath (Duiven, The Netherlands). All enzymes used for protein digestibility analysis, including trypsin, chymotrypsin, and protease, were acquired from Sigma-Aldrich.

### 2.2 Sample Preparation and Milling

Both Ramon fruits and seeds were prepared and analyzed in this study. The selected matured fruits (FR) with an average weight of 5 kg were peeled to obtain the seeds, which were then washed with tap water and dried under the sun for up to 2 h to remove any excess water (Figure S1). These fruits were freeze-dried (Labconco Freeze-Dryer, Kansas City, USA) prior to their subsequent grinding procedures. A total of half of the seeds was designated for green seed (GS) samples, while the remaining half was selected for use as roasted samples (RS). The coating layer was separated from the green seed samples before they were cut into four parts to enhance the grinding process, afterwards they were freeze-dried. All chemical and proximal analyses described below were performed on freeze-dried and milled powders obtained from Ramón seeds and fruits. Conversely, in the case of the roasted seeds, the seeds with their coating layer intact were subjected to roasting using a vacuum oven (BINDER vacuum drying chambers, Lakeville, USA) at 60 °C for 72 h, and upon completion of the heating process, the roasted seeds were collected as a sample (RS), while their coating layer was taken as another type of sample (SC). All the samples were then milled using a cryogrinder equipment (Fritsch P11 Knife Mill, Winchester, UK) with liquid nitrogen to prevent heating and preserve the volatile components of the seeds, and avoid fat oxidation and Maillard reactions. The resulting flours were sieved using a laboratory test sieve with a mesh size of 250 μm (60 mesh), and the homogeneous flours obtained were stored at room temperature for further analyses.

### 2.3 Proximal Evaluation of Ramon Seeds

#### 2.3.1 Proximate Composition

The proximate composition of green Ramon Seeds (GS), roasted Ramon seeds (RS), fruit of Ramon (FR), and seed coat layer of Ramon seeds (SC) flour was assessed following the official methods of the American Association of Cereal Chemists.^14^ Ash (AACC 08-16.01) and lipid (AACC 30-25.01) contents were determined using standard procedures. Protein content was measured by the Kjeldahl method (AACC 46-30.01), and crude protein was calculated using a nitrogen-to-protein conversion factor of 6.25. All determinations were performed in quintuplicate, and the mean values were reported.

#### 2.3.2 Total Dietary Fiber, Available Carbohydrates, and Resistant Starch

Total dietary fiber (soluble and insoluble fractions), as well as available carbohydrates, were quantified using the Megazyme kit (K-ACHDF 06/18), following AOAC (Official Method 991.43)^15^ and AACC (Method 32-07.01)^16^ protocols, according to the manufacturer’s instructions. Calculations were carried out with the Mega-Calc tool (K-TDFR) provided by Megazyme.^17^

#### 2.3.3 Available Carbohydrates

Available carbohydrates (d-glucose and D-fructose) were determined from aliquots prepared during the dietary fiber analysis, following the manufacturer’s protocol. The results were calculated using the Mega-Calc spreadsheet (K-ACHDF).^18^

#### 2.3.4 Resistant Starch

Resistant starch (RS) and nonresistant starch (NRS) were analyzed using the Megazyme kit (K-RAPRS 11/19), based on AOAC (Official Method 2002.02)^19^ and AACC (Method 32-40)^20^ procedures, with minor modifications. The quantification of RS and NRS was performed using the Mega-Calc software (K-RAPRS), available from ref 21.

### 2.4 Minerals Quantification

The method used for mineral quantification was adapted from Kendall et al.^22^ Dried samples were weighed into a high-pressure digestion vessel (HVT50) to which 3 mL of 68% nitric acid, 3 mL of deionized water, and 2 mL of 30% hydrogen peroxide were added prior to processing in a microwave digestion system (Multiwavepro) featuring a 10 min ramp to 140 °C, a 20 min maintenance period at 140 °C, and subsequent cooling to 55 °C. The digested contents were then transferred to a 25 mL universal tube with 7 mL of deionized water. Blanks and suitable standards, and certified reference materials were incorporated with each analytical batch. Multielement analysis (Ca, P, Mg, Na, K, Cu, S, Fe, Mo, Mn, Pb, Cd, As, B, Al, Ni, Se, Co, Zn) was taken after the dilution of 1:20 (0.5 mL:10 mL) with 0.5% nitric acid. This was via an inductively coupled plasma mass spectrometer (ICP-MS) (Thermo-Fisher iCAP-Q) equipped with a “Flatopole collision cell”. Internal standards were integrated into the sample stream through a T-piece and comprised Sc, Ge, Rh, and Ir within a matrix of 2% HNO_3_. External calibration standards were typically maintained within the range of 0–100 μg/L^–1^ (ppb) for trace elements and 0–100 mg L–1 (ppm) for macro elements. Samples were introduced via a covered autosampler (Cetac ASX-520) through a 1317090 pfa-st nebulizer (ESI). Sample processing was executed by utilizing “Qtegra software”.

### 2.5 Antinutritional Factors (ANFs) of different components of Ramon Seeds

#### 2.5.1 Total Phenolics

Total phenolic content was determined via the Folin-Ciocalteu and Fast Blue BB assays.^23^ The sample was first extracted as per Pico et al.,^24^ with the following modifications. Total extractable phenolic compounds (EPP), also defined as free phenolic compounds (samples with the polar interferences act as a control), were extracted initially with 80% methanol in 0.1% formic acid, followed by the addition of 2% EDTA. About 70% acetone in 0.1% formic acid was then used for a second extraction process. The extracted sample was stored at −80 °C prior to analysis or further purification by solid-phase extraction (SPE) to remove polar interferences and produce a refined extract.

Physical removal of polar interferences from the EPP extract by SPE was determined to compare the results of total phenolic compounds with the control sample when using both assays. Oasis HLB 1 cm^3^ (30 mg) cartridges were used by following the protocol from Manzanilla-Valdez et al.^25^ with slight modifications. The final extractable compounds from the SPE were used for further analysis with the Folin-Ciocalteu and Fast Blue BB assays.

##### 2.5.1.1 Folin–Ciocalteu Assays

Each extracted sample (EPP and SPE) and gallic acid standard were mixed with Folin reagent and 4% Sodium carbonate solution in a 96-well plate. The sample was incubated for 30 min at room temperature in the dark, and the spectrophotometric determination was performed at 765 nm using Tecan Austria GmbH (Model SPARK 10M, Berkshire, UK). The absorbance values were interpolated in the standard curve of gallic acid (0–500 μg of gallic acid/mL), and then the μg/mL of gallic acid in samples was calculated and expressed as milligram equivalent of gallic acid per 100 g of sample. All analyses were carried out in triplicate.^23^

##### 2.5.1.2 Fast Blue BB Assays

A solution containing 0.1% Fast Blue BB reagent and 5% NaOH was added to each extracted sample (from both EPP and SPE) and to each standard in a 96-well plate. The sample was then incubated for 2 h before being measured at an absorbance of 420 nm using Tecan Austria GmbH. The absorbance values were interpolated in the standard curve of gallic acid (0–500 μg/mL of gallic acid/mL), and then the μg/mL of gallic acid in samples was calculated and expressed in milligrams of gallic acid equivalents (GAE) per 100 g of sample. All analyses were carried out in triplicate.^23^

#### 2.5.2 Saponins

The method was adapted from Hiai et al.^26^ by using spectrophotometry. The sample was extracted with 80% of methanol and quantified via the “vanillin-sulfuric assay” method based on the reaction between sulfuric acid-oxidized triterpene saponin with vanillin. An orange-purple color appears after the reaction occurs, and the intensity of this color change can be measured at a wavelength of 560 nm. Total saponin content was calculated from a standard curve of diosgenin (0–0.5 mg/mL) and was expressed as mg of diosgenin equivalents (mg DE/100 g).^27^

#### 2.5.3 Tannins

Tannins were extracted by mixing 0.5 g of Ramon samples and 5 mL of 4% HCl in methanol for 18 h.^28^ The samples were then centrifuged at 4500*g* for 10 min, and the supernatants were collected. In wells of a 96-well plate, 50 μL of sample extract, 100 μL of 1% vanillin in methanol, and 100 μL of 10% HCl in methanol were added and incubated for 10 min at RT. The absorbance was determined at 500 nm using a microplate reader (Infinite 200 PRO, Tecan Trading AG, Männedorf, Switzerland). Catechin was used as a standard (0.25–1.0 mg/mL), and the tannin content was expressed as mg of catechin equivalent per gram of sample (mg CE/g).

#### 2.5.4 Phytic Acid

The assay was determined using the colorimetric method as described by Latta and Eskin^29^ and modified by Gao et al.^30^ Phytate was extracted using 2.4% HCl (w/v), and the crude extracts obtained were mixed with 1 g of NaCl, dissolved, and incubated to precipitate the matrix components. The diluted samples were then treated with modified Wade reagent (0.03% FeCl_3_·6H_2_O, 0.3% sulfosalicylic acid). A phytic acid standard was also prepared and treated the same way as described above. The phytate content was then measured at 500 nm using a Tecan Austria GmbH and expressed as milligrams of phytic acid equivalent per 100 g of sample (PAE g/100 g). The phosphorus content of sodium phytate was 18.38% and accounted for in the calculation of the phytate content.^31^

#### 2.5.5 Oxalates

The oxalate content was determined by the method described by Ukpabi and Ejidoh^32^ with several modifications. The procedure involves three main steps, including digestion, oxalate precipitation, and permanganate titration. The sample was digested with 6 M HCl in a hot water bath and filtered. The filtrate was then added with the methyl red indicator and concentrated NH_4_OH solution until a faint yellow color was obtained (pH 5.6–6.2). It was then incubated at 90 °C and filtered, and 5% CaCl_2_ solution was added before incubation at room temperature overnight. The solution was centrifuged, and the precipitate was dissolved in a 20% (v/v) H_2_SO_4_ solution. The final precipitate was heated near the boiling point and then titrated against 0.05 M KMnO_4_ solution until a faint pink color persisted for 30 s. The final volume of titration was recorded, and the calcium oxalate content was calculated based on the formula:^33^

$$\mathrm{Calcium}\;\mathrm{oxalates}=\frac{T\times (V_{\mathrm{me}})\times \mathrm{DF}\times 10^{5}}{(\mathrm{ME})\times m_{f}}$$

where *T* = titer of KMnO_4_ (mL), *V*_me_ = volume mass equivalent (i.e., 1 cm^3^ of 0.05 M KMnO_4_ solution is equivalent to 0.00225 g anhydrous oxalic acid), DF = dilution factor, *V*_T_ *A* (2.4, where *V*_T_ is the total volume of the filtrate (300 mL) and *A* is the aliquot used (125 mL)), ME = molar equivalent of KMnO_4_, in oxalate (KMnO_4_, redox reaction (×10^5^)) and *m*_f_ is the mass of flour used.

#### 2.5.6 Trypsin-Inhibitory Activity

The method was adapted from the American Oil Chemists’ Society (AOCS) Method Ba12a-2020. Ground and sieved samples (<300 μm diameter) were extracted with 10 mM NaOH. The extracted sample was diluted with distilled water to obtain a dilution whereby 1 mL of extract produced a trypsin inhibition activity of between 30–60%. Each diluted sample was mixed with BAPA and trypsin solution in a 37 °C water bath. The reaction was stopped after 10 min by the addition of 30% acetic acid. The reference sample (Milli-Q water) was also used to measure the trypsin inhibitory activity. The absorbance was read at 410 nm against the sample blank. The trypsin inhibitory activity (TIA) was calculated as follows:^34^

$$\mathrm{TIA}\;(\%)=1-\left(\frac{\left(\mathrm{Absorbance}\;\mathrm{of}\;\mathrm{sample}-\mathrm{Absorbance}\;\mathrm{of}\;\mathrm{sample}\;\mathrm{blank}\right)}{\left(\mathrm{Absorbance}\;\mathrm{of}\;\mathrm{reference}-\mathrm{Absorbance}\;\mathrm{of}\;\mathrm{reference}\;\mathrm{blank}\right)}\right)\times 100$$

One TU is considered as an increase of 0.02 absorbance at 410 nm. TIA is expressed as TU inhibited (TUI) (also known as trypsin inhibitor units, TIU in some literature) per milligram of sample and can be calculated by the following equation:

$$\mathrm{TUI}\;(\mathrm{mg}\;\mathrm{of}\;\mathrm{sample})=\frac{\Delta \mathrm{Absorbance}\times 50}{\mathrm{mg}\;\mathrm{of}\;\mathrm{sample}}$$

where

$$\Delta \mathrm{Absorbance}=\frac{\left(\mathrm{Absorbance}\;\mathrm{of}\;\mathrm{reference}-\mathrm{Absorbance}\;\mathrm{of}\;\mathrm{reference}\;\mathrm{blank}\right)}{\left(\mathrm{Absorbance}\;\mathrm{of}\;\mathrm{sample}-\mathrm{Absorbance}\;\mathrm{of}\;\mathrm{sample}\;\mathrm{blank}\right)}$$

#### 2.5.7 Anthocyanins

The total monomeric anthocyanin pigment content was determined using AOAC Official Method 2005.02, which involved extracting the sample with acidified methanol (4% HCl in MeOH), applying a suitable dilution factor to achieve an absorbance within the spectrophotometer’s linear range at 520 nm, and subsequently preparing two dilutions with pH 1.0 and pH 4.5 buffers. Absorbance measurements of these diluted samples and buffers were taken at 520 and 700 nm, using distilled water as a blank, and the anthocyanin pigment concentration was calculated and expressed as cyanidin-3-glucoside equivalents per liter (C3GE/L).^35^

#### 2.5.8 Hemagglutination Activity

##### 2.5.8.1 Lectin Extraction

Lectin extraction was performed according to the methodology of Mejia et al.,^36^ with some modifications. Extraction was carried out by overnight incubation of Ramon seed flour 1:10 (w/v) in 10 mM phosphate-buffered saline (PBS), pH 7.4, at 4 °C. Then, the solution was centrifuged at 12,000*g* at 4 °C for 30 min before being brought to 80% (NH_4_)_2_SO_4_ saturation. Stirring for 1 h at 4 °C is recommended to make sure the salt has been fully dissolved. The protein was then obtained by centrifugation at 12,000*g* at 4 °C for 30 min. The precipitate was resuspended in PBS (1:10, w/v), dialyzed overnight using a 30 mL Thermo Scientific Slide-A-Lyzer G2 Dialysis Cassette with a 10K molecular weight cutoff against distilled water, and then lyophilized for further use.

##### 2.5.8.2 Hemagglutination Assay

The sheep hemagglutination kit was used to perform this assessment. The test was carried out using Nunc 96-Well Polystyrene Round Bottom Microwell Plates (Thermo Fisher Scientific, Waltham, MA, USA). The lectin extract obtained from the previous step was diluted serially, adjusting the sample volume in each well to 50 μL with PBS (as a negative control), 50 μL of each prepared dilution (both antibody and sample, including positive control), and finally 50 μL of a 0.5% RBC stock solution were added into each well. The plate was gently moved in circles on a flat surface for 10 s and incubated for 90 min at room temperature. The plate was imaged using a lit background with a phone camera or an equivalent for subsequent analysis.

### 2.6 Total Amino Acids

About 2 mg of the different components of Ramon seed, including the seed coat layer of the seeds (SC), fruit of Ramon (FR), and both roasted (RS) and green (GS) (unprocessed) seeds, were hydrolyzed in 6 N HCl (4 mL) at 110 °C for 24 h in tubes sealed under nitrogen. Amino acids were determined after derivatization with diethyl ethoxymethylenemalonate by HPLC according to the method of Alaiz et al.,^37^ using D,L-α-aminobutyric acid as an internal standard and a 300 mm × 3.9 mm i.d. Reversed-phase column (Novapack C18, 4 μm; Waters, Milford, MA, USA). Tryptophan was analyzed by HPLC after basic hydrolysis according to Yust et al.^38^

### 2.7 Protein Characterization

#### 2.7.1 Sodium Dodecyl Sulfate-Polyacrylamide Gel Electrophoresis (SDS-PAGE) Analysis

The molecular weight of the different components of Ramon seed, including the seed coat layer of the seeds (SC), fruit of Ramon (FR), and both roasted (RS) and green (GS) (unprocessed) seeds, was analyzed by SDS-PAGE.^39^ Dual Xtra standard (2–250 kDa, Bio-Rad, CA, USA) was used as a standard marker. Fifteen μg of protein was mixed with Laemmli buffer (0.1 M Tris-Tricine, pH 6.8, 2% SDS, and 0.025% bromophenol blue, as well as 5% β-mercaptoethanol for reducing conditions) and boiled for 5 min before loading into each well. Proteins were separated using 12% Criterion XT Bis/Tris precast gels (18-well, 1.0 mm thickness) with MES SDS running buffer (50 mM MES, 50 mM Tris–HCl, 0.1% SDS, 1 mM EDTA; Bio-Rad, Hercules, CA, USA) in a Criterion vertical electrophoresis cell (Bio-Rad). Lastly, the SDS gels were stained using Biosafe Coomassie Brilliant Blue and analyzed by gel imager system (Gel Doc XR + system, Bio-Rad). The molecular weight of the bands was determined using Image Lab software (Image Lab 6.1, BIO-RAD).

#### 2.7.2 In-Gel Digestion and LC–MS/MS Identification

The most prominent bands from Ramon seeds were excised for in-gel digestion and subsequent proteome analysis by LC-MS/MS acquisition at the University of York. Peptide mixture analyses were conducted using a Vanquish Neo UHPLC system linked to an Orbitrap Eclipse Tribrid mass spectrometer (Thermo Fisher Scientific, Sunnyvale, CA, USA). Before LC separation, tryptic digestates were concentrated and desalted using a trapping column (300 μm × 5 mm, μPrecolumn, 5 μm particles, Acclaim PepMap100 C18, Thermo Fisher Scientific) at ambient temperature (20 °C). Following the washing of the trapping column with 0.1% formic acid, the peptides were eluted (flow rate–0.25 nL/min) from the trapping column to an analytical column (EASY spray, 2 μm particles, 75 μm × 500 mm, Thermo Fisher Scientific) at 45 °C using a 75 min linear gradient program (2–40% of mobile phase B; mobile phase A: 0.1% formic acid in water; mobile phase B: 0.1% formic acid in 80% acetonitrile). The equilibration of both the trapping column and the analytical column was conducted immediately before sample injection. The analytical column with the emitter was directly linked to the ion source. Mass spectrometry data were obtained by using a data-dependent approach. The survey scan range was established at *m*/*z* 350–2000, with a resolution of 120,000 (at *m*/*z* 200), a target value of 3 × 10^6^ ions, and a maximum injection time of 50 ms. Higher-energy collisional dissociation (HCD) MS/MS spectra were obtained using a normalized fragmentation energy of 30%, a maximum injection duration of 54 ms, and a resolution of 30,000 at *m*/*z* 200. Dynamic exclusion was activated for 60 s. The isolation window for MS/MS fragmentation was established at 1.2 *m*/*z*.

Progenesis QI (v4.2, Waters) software was used for peak picking, chromatographic alignment, and export of a concatenated peak list in.mgf format. Data were searched using Mascot (v2.8.3, Matrix Science) against the *Morus* subset of UniProt (Ver, 07 May 2024, 28,360 sequences) appended with common proteomic contaminants. Search criteria specified: Enzyme, Trypsin; Fixed modifications, Carbamidomethyl (C); Variable modifications, Acetyl (Protein N-term), Oxidation (M); Mass values, Monoisotopic; Peptide mass tolerance, 10 ppm; Fragment mass tolerance, 0.02 Da; and Max missed cleavages, 1. Results were filtered to 1% FDR as assessed empirically against a reversed database using the Percolator algorithm and required a minimum of two unique and three total peptides per accepted protein. Matches to contaminant proteins were stripped from the results. Accepted peptide identifications were exported from Mascot in.xml format and imported into Progenesis QI, and identifications were mapped between runs. Peak areas were integrated, and a Top 3 approach was applied for estimation of relative protein abundance. Estimations of molar abundance were calculated by expressing each Top 3 value as a proportion of the sum of all values.

### 2.8 Protein Quality Assessment

#### 2.8.1 Amino Acid Score

The amino acid score (AAS) was estimated using the following equation with the reference pattern of the FAO report 1985 (FAO/WHO/UNU, 1985):

$$\mathrm{AAS}=\frac{\mathrm{mg}\;\mathrm{of}\;\mathrm{amino}\;\mathrm{acids}\;\mathrm{in}\;1\;g\;\mathrm{of}\;\mathrm{total}\;\mathrm{protein}}{\mathrm{mg}\;\mathrm{of}\;\mathrm{amino}\;\mathrm{acids}\;\mathrm{in}\;1\;g\;\mathrm{requirement}\;\mathrm{protein}}\times 100$$

#### 2.8.2 Essential Amino Acid Index (EAAI)

The essential amino acid index (EAAI) was calculated using the amino acid composition of a standard (whole egg protein):^40^

$$\mathrm{EAAI}=\sqrt[{9}]{\frac{(\mathrm{Lys}\times \mathrm{Thr}\times \mathrm{Val}\times (\mathrm{Met}+\mathrm{Cys})\times \mathrm{Ile}\times \mathrm{Leu}\times (\mathrm{Phe}+\mathrm{Pro})\times \mathrm{His}\times \mathrm{Trp})a}{(\mathrm{Lys}\times \mathrm{Thr}\times \mathrm{Val}\times (\mathrm{Met}+\mathrm{Cys})\times \mathrm{Ile}\times \mathrm{Leu}\times (\mathrm{Phe}+\mathrm{Pro})\times \mathrm{His}\times \mathrm{Trp})b}}\times 100$$

where *a* = the content of amino acids in the sample and *b* = the content of similar amino acids in the standard, EAAI is expressed in %.

#### 2.8.3 Predicted Biological Value (BV)

The predicted biological value (BV) was calculated according to Amza, A^40^ using the following equation:

BV=1.09(EAAI)−11.7

#### 2.8.4 Protein Efficiency Ratio (PER)

The protein efficiency ratio was calculated as follows by using five equations with the values obtained from the amino acid composition of Ramon samples:^40^

$${\mathrm{PER}}_{1}=-0.684+0.456(\mathrm{Leu})-0.047(\mathrm{Pro})$$

$${\mathrm{PER}}_{2}=-0.468+0.454(\mathrm{Leu})-0.105(\mathrm{Tyr})$$

$${\mathrm{PER}}_{3}=-1.816+0.435(\mathrm{Met})+0.780(\mathrm{Leu})+0.211(\mathrm{His})-0.944(\mathrm{Tyr})$$

$${\mathrm{PER}}_{4}=0.08084(\mathrm{Thr}+\mathrm{Val}+\mathrm{Met}+\mathrm{Ile}+\mathrm{Leu}+\mathrm{Phe}+\mathrm{Lys})-0.1094$$

$${\mathrm{PER}}_{5}=0.0632(\mathrm{The}+\mathrm{Val}+\mathrm{Met}+\mathrm{Ile}+\mathrm{Leu}+\mathrm{Phe}+\mathrm{Lys}+\mathrm{His}+\mathrm{Arg}+\mathrm{Tyr})-0.1539$$

### 2.9 In Vitro Protein Digestibility

The *in vitro* protein digestibility was performed using the pH-drop method adapted from Tinus et al.,^41^ in which 62.5 ± 0.5 mg protein of each sample was resuspended in 10 mL DI H_2_O and adjusted to a pH of 8.0. Subsequently, the chymotrypsin (31 mg), trypsin (16 mg), and protease (13 mg) enzyme cocktail with an adjusted pH of 8.0 was added to the protein solution. The pH of the digesta was recorded every 30 s for 10 min. The change in pH at 10 min (ΔpH_10min_) of digestion was used to calculate the percentage of in vitro protein digestibility (IVPD) of the sample as follows:

$$\mathrm{IVPD}=65.66+18.10\times \Delta {\mathrm{pH}}_{10\min}$$

where (ΔpH_10min_) is the change in pH from the initial pH of about 8.0.

Meanwhile, for the *in vitro* protein-digestibility corrected amino acid score (IVPDCAAS), it was calculated as a product of the AAS and IVPD.^42^

### 2.10 Gluten Analysis

The gluten content of the sample was analyzed using the Wheat/Gluten (Gliadin) ELISA Kit (Crystal Chem, USA). Gluten proteins were first extracted from the sample by overnight incubation. The extracted gliadin was then bound to polyclonal antibodies precoated onto the surface of a microplate. Following this, incubation and washing steps were carried out to remove the unbound material. An enzyme conjugate was subsequently added to form an antibody–antigen–enzyme complex on the plate surface. A substrate solution was then added to initiate a colorimetric reaction, which was stopped by the addition of 1 N sulfuric acid. Absorbance was measured at 450 nm with a reference wavelength of 630 nm by using a microplate reader. The gluten protein content in the sample was interpolated from a standard calibration curve. A conversion factor of 0.85 was applied to estimate the total gluten content based on gliadin concentration.

### 2.11 Statistical Analysis

All experiments were performed in triplicate, except for mineral analysis and total amino acid determination, which were conducted in duplicate. Data were expressed as the mean ± the standard deviation. Where applicable, the data were examined by using analysis of one-way analysis of variance (ANOVA). The significant differences were determined at a probability level of 0.05 by the Tukey test, and a *p*-value less than 0.05 was considered statistically significant.

## 3 Results and Discussion

### 3.1 Nutritional Composition of different components of Ramon Seeds

The nutritional compositions of the different Ramon fractions are presented in Table 1. The nutritional composition presented are in dwb in g/100 g of sample, except for dietary fiber, presented as a percentage value.

**Table 1:** Nutritional Composition of Green Ramon Seeds (GS), Roasted Ramon Seeds (RS), Fruit of Ramon (FR), Seed Coat Layer of Ramon Seeds (SC) Flours (Dry Basis)[Table-fn t1fn1]

Nutrient	GS	RS	FR	SC
Protein (g/100 g)[Table-fn t1fn2]	10.69 ± 0.01^a^	10.65 ± 0.01^a^	9.85 ± 0.11^a^	10.53 ± 0.24^a^
Fat (g/100 g)	1.35 ± 0.02^c^	1.99 ± 0.07^b^	0.92 ± 0.12^c^	3.16 ± 0.19^a^
Ash (g/100 g)	1.61 ± 0.05^b^	1.89 ± 0.02^b^	2.36 ± 0.01^a^	2.24 ± 0.01^a^
Moisture (g/100 g)	4.28 ± 0.07^b^	6.20 ± 0.03^a^	4.99 ± 0.01^c^	4.39 ± 0.05^d^
Available carbohydrates (g/100 g)				
d-glucose (g/100 g)	59.75 ± 0.12^a^	51.24 ± 0.35^b^	21.40 ± 0.35^c^	6.65 ± 0.18^d^
D-fructose (g/100 g)	0.92 ± 0.24^b^	0.37 ± 0.06^b^	19.44 ± 0.24^a^	0.17 ± 0.12^b^
Total available carbohydrates (g/100 g)	60.67 ± 0.36^a^	51.61 ± 0.41^b^	40.84 ± 0.59^c^	6.82 ± 0.30^d^
Dietary fiber (%) (w/w)				
Insoluble fiber (%) (w/w)	14.47 ± 0.06^d^	17.88 ± 0.12^c^	27.42 ± 0.18^b^	71.96 ± 0.97^a^
Soluble fiber (%) (w/w)	1.02 ± 0.01^c^	1.61 ± 0.01^b^	2.21 ± 0.01^a^	0.87 ± 0.04^d^
Starch (g/100 g)				
Resistant starch (g/100 g)	8.30 ± 0.21^a^	1.01 ± 0.05^c^	0.74 ± 0.01^c^	3.99 ± 0.27^b^
Non-resistant starch (g/100 g)	0.77 ± 0.03^c^	1.16 ± 0.01^b^	2.53 ± 0.05^a^	0.40 ± 0.02^d^

* Different lowercase letters (a−d) within each row indicate significant differences (*p*-value <0.05), Tukey's test. *N* = 3 for all variables. GS: Green Ramon Seeds, RS: Roasted Ramon Seeds, FR: Fruit of Ramon, SC: Seed coat layer of Ramon seeds. All data presented are based on dry weight basis (g/100 g) except for dietary fiber which is presented in percentage.

** Protein = *N* × 6.25 (16% nitrogen in protein).

In this study, available carbohydrates were defined as the sum of free sugars and complex carbohydrates that are digested and absorbed by the human body.^43^ GS exhibited a significantly higher available carbohydrate content (60.67 g/100 g) compared to the RS (51.61 g/100 g) (*p* < 0.05). This difference was likely due to the Maillard reaction, where chemical interactions between amino acids and reducing sugars occur during high-temperature processing or cooking, resulting in the formation of brown pigments and distinct flavours.^44^ However, the carbohydrate values reported in this study are considerably lower than those reported by Carter and Northcutt^9^ and Losoya et al.,^10^ who found carbohydrate contents of 83.61 g/100 g for unroasted seeds and 82.61–84.67 g/100 g for roasted seeds, on a dry weight basis. These differences may be explained by differences in other nutritional parameters such as crude protein, fat, moisture, and ash, which were reported to be slightly higher in their studies. Currently, there is limited information about the chemical composition of FR or its individual anatomical components (seed coat layer). Compared with other Moraceae fruits, FR exhibited a higher carbohydrate content (43.01 g/100 g). For example, Figs (*Ficus carica*) contain 19.2 g/100 g, jackfruit (*Artocarpus heterophyllus*) 16.0–25.4 g/100 g, and breadfruit (*Artocarpus altilis*) 22.8 g/100 g.^45–47^ Relative to fruits commonly consumed in the Yucatán Peninsula such as papaya (*Carica papaya*) with >9.5 g/100 g, and mamey sapote (*Pouteria sapota*) with >31.1 g/100 g,^48,49^ Ramón also shows a higher carbohydrate level. Therefore, Ramón fruit can be regarded as a carbohydrate source comparable to and, in some respects, superior to other Moraceae species and commonly consumed regional fruits.

Protein content of RS was not significantly different (10.65%) from that of GS (10.69%). However, the FR had a significantly lower amount of protein content (9.85%, *p* < 0.05) compared to other fractions studied. The protein content found in this study falls within the range reported in previous publications, with Ramon seed flour ranging between 9.94–11.74% and roasted seed flour from 9.5–12.14%.^2,3,9^ Interestingly, the seed coat layer (SC) had a protein content almost similar to that of the whole seeds, indicating that protein is also present in the seed coat.

Fat content was higher in the SC (3.16%) compared to the whole seeds (1.35%). A statistically significant difference (*p* < 0.05) was also observed between the fat content of GS (1.35%) and RS (1.99%). The results contrast with those reported by Carter and Northcutt,^9^ who found no significant difference in fat content between roasted (0.64 g/100 g) and unroasted seeds (0.58 g/100 g). Higher amounts of resistant starch were found in GS (8.30 g/100 g), likely due to their higher carbohydrate content. Resistant starch is a form of non-digestible carbohydrate that escapes digestion in the small intestine and may confer health benefits.^50^ In comparison, FR showed a higher content of non-resistant starch (2.53 g/100 g) compared to other seed components.

Among all fractions analyzed, SC had the highest total dietary fiber content (72.83%). This finding aligns with histochemical studies on*Brosimum alicastrum* species conducted in Mexico by Brechú-Franco et al.,^51^ which identified a lignified layer in mature seeds composed of several layers of tanniferous cells and a coriaceous membrane rich in fibers and vascular bundles in the hilar region—factors that likely contribute to the high fiber content. Insoluble fiber accounted for 71.86% of the total dietary fiber in SC. Similar findings have been reported in pulse seed coats, such as those of chickpea, which contain 49.1–52.9 g/100 g insoluble fiber and 1.9–2.5 g/100 g soluble fiber.^52^

Moisture content was highest in RS (6.20 g/100 g) among all fractions. This is because roasted samples did not undergo freeze-drying, unlike the GS samples. The moisture values reported in this study are slightly lower than those reported in previous studies. All nutritional composition data presented in this section refer to the freeze-dried, milled powders prepared from Ramon seeds and fruits. This approach was selected because the lyophilized powders are the intended material for downstream nutritional and functional applications; removal of water concentrates main constituents and improves sample stability, thereby facilitating more accurate and reproducible measurements. For instance, Carter and Northcutt^9^ reported higher moisture levels in unroasted seed flour (11.75%) compared to roasted seed flour (8.78%). However, similar values were observed by Losoya et al.,^10^ who reported moisture contents of 6.05 and 5.92% for Maya nut flours dried at 45 and 60 °C, respectively. These differences may be attributable to multiple factors, including varietal (genetic) differences, agronomic and environmental conditions (e.g., climate), postharvest handling, analytical methodologies, and the drying protocols employed.

Ash refers to the inorganic residue remaining after the complete incineration of water and organic matter and serves as an indicator of the total mineral content in a food sample. In this study, the ash content was slightly lower than values reported in previous studies, with GS showing the lowest ash content (1.61 g/100 g). Quintero-Hilario et al.^3^ reported ash contents of 3.22% in fresh seeds and 3.53–3.57% in roasted seeds, depending on the specific roasting conditions. The higher ash content observed in SC (2.24 g/100 g) suggests that this layer contains a greater concentration of minerals compared to other seed fractions.

### 3.2 Mineral Content

The Ramon seeds were found to contain high levels of essential minerals, including Ca, Mg, K, and P (Table 2). The SC exhibited higher concentrations of most minerals, which aligns with the findings from the ash content analysis. Notably, the concentrations of key minerals in GS were slightly lower than those reported by Losoya et al.,^10^ who reported values of 829.08 mg/100 g for Ca and 2608.81 mg/100 g for K, compared to 108.99 mg/100 g and 1429.95 mg/100 g, respectively, in the present study. These discrepancies could be attributed to differences in analytical techniques, sample preparation methods, and geographic origin, all of which can influence the mineral composition.

**Table 2:** Mineral Content of Green Ramon Seeds (GS), Roasted Ramon Seeds (RS), Fruit of Ramon (FR), and Seed Coat Layer of Ramon Seeds (SC) Flours (Expressed in mg/100 g)[Table-fn t2fn1]

Minerals	GS	RS	FR	SC
**Essential minerals**				
Zn	1.16 ± 0.03^b^	1.26 ± 0.02^b^	1.39 ± 0.06^b^	3.39 ± 0.18^a^
Fe	1.86 ± 0.27^b^	2.6 ± 0.27^b^	3.53 ± 0.24^b^	40.12 ± 4.93^a^
Ca	108.99 ± 3.72^b^	168.84 ± 4.96^b^	389.87 ± 2.31^c^	1,244.33 ± 83.16^a^
Na	5.26 ± 0.21^b^	6.54 ± 0.47^b^	55.48 ± 1.97^c^	243.48 ± 11.36^a^
Mn	0.35 ± 0.02^b^	0.44 ± 0.01^b^	0.53 ± 0.01^bc^	4.55 ± 0.05^a^
Cl	12.39 ± 1.65^b^	43.92 ± 5.13^b^	77.72 ± 10.42^bc^	296.00 ± 18.33^a^
Mg	214.35 ± 13.44^b^	207.57 ± 11.06^b^	189.06 ± 4.38^b^	314.69 ± 23.61^a^
K	1429.95 ± 48.64^b^	1427.61 ± 27.80^b^	2105.27 ± 14.71^a^	914.88 ± 44.72^c^
P	187.69 ± 14.40^ab^	196.56 ± 7.24^ab^	209.24 ± 0.31^a^	128.08 ± 10.33^c^
Cu	0.46 ± 0.02^b^	0.60 ± 0.01^bc^	0.45 ± 0.01^b^	1.48 ± 0.08^a^
Se	0.02 ± 0.01^a^	0.01 ± 0.01^b^	0.01 ± 0.01^c^	0.01 ± 0.01^c^
S	0.12 ± 0.03^a^	0.12 ± 0.01^a^	0.09 ± 0.01^a^	0.16 ± 0.06^a^
**Non-essential minerals**				
Pb	0.03 ± 0.01^b^	0.01 ± 0.01^bc^	0.01 ± 0.01^c^	0.11 ± 0.01^a^
Cd	0.01 ± 0.01^b^	0.02 ± 0.01^b^	0.02 ± 0.01^c^	0.02 ± 0.01^a^
B	0.73 ± 0.16^b^	0.74 ± 0.31^b^	1.50 ± 0.10^c^	2.68 ± 0.13^a^
Al	0.68 ± 0.06^b^	0.58 ± 0.19^b^	2.87 ± 0.17^b^	73.08 ± 7.94^a^
As	0.01 ± 0.01^b^	0.02 ± 0.01^b^	0.02 ± 0.01^b^	0.02 ± 0.01^a^
Ni	0.17 ± 0.01^b^	0.12 ± 0.01^bc^	0.07 ± 0.01^c^	0.18 ± 0.03^ab^
Mo	0.07 ± 0.01^b^	0.09 ± 0.01^bc^	0.10 ± 0.01^c^	0.28 ± 0.01^a^

* Different lowercase letters (a−d) within each row indicate significant differences (p-value <0.05), Tukey test. *N* = 3 for all variables. GS: Green Ramon Seeds, RS: Roasted Ramon Seeds, FR: Fruit of Ramon, SC: Seed coat layer of Ramon seeds.

### 3.3 Antinutritional Composition of Ramon Seeds Components

Phenolic compounds are known to influence various sensory and functional properties of food, including bitterness, astringency, color, flavor, odor, and oxidative stability.^53^ They also exhibit numerous beneficial biological effects, with antioxidant capabilities being the most extensively studied. However, it is crucial to understand that phenolic compounds can interact with other components of the food matrix, such as proteins, carbohydrates, and lipids, which can influence their bioavailability and functional properties.^54,55^ The formation of protein-phenolic complexes may significantly impact protein structure, potentially affecting both protein digestibility and phenolic activity.^55^ In this study, the total phenolic content was measured using two different analytical methods: the Folin–Ciocalteu (FC) and Fast Blue BB (FBBB) assays (Table 3). Considering both methods is important, as they complement each other. The FC assay provides a broad estimate of total reducing capacity since it responds not only to phenolic compounds but also to other reducing agents such as sugars or ascorbic acid, which may lead to overestimation. In contrast, the FBBB method is a diazonium coupling reaction that targets phenolic–OH groups under alkaline conditions, forming stable azo chromophores (typically read at ∼420 nm). Because it largely ignores nonphenolic reducers, FBBB is generally more selective and often more accurate for estimating true phenolic content. By applying both assays, a more reliable and nuanced assessment of phenolic compounds in plant-based foods can be achieved, while also accounting for possible matrix-dependent interferences.^56^

**Table 3:** Extractable Phenolic Compounds (EPP) Measured by Folin-Ciocalteu and Fast Blue BB (FBBB) Assays (Expressed in mg/100 g of Gallic Acid Equivalents) without the Removal of Interferences (Control) and after the Removal of Soluble Interferences by Solid Phase Extraction (SPE)[Table-fn t3fn1],[Table-fn t3fn2]

	Folin-Ciocalteu			Fast Blue BB		
Samples	Control (EPP)	Solid phase extraction (SPE)	↑↓ (%)	Control (EPP)	Solid phase extraction (SPE)	↑↓ (%)
**GS**	2781.26 ± 14.22^Ba^	2883.20 ± 4.85^Ab^	↑ (3.66)	1290.00 ± 5.09^Dc^	2427.00 ± 0.28^Cb^	↑ (88.14)
**RS**	2326.17 ± 20.80^Bb^	2933.94 ± 18.75^Aa^	↑ (26.13)	729.20 ± 11.31^Dd^	1178.87 ± 18.20^Cd^	↑ (61.67)
**FR**	881.58 ± 2.72^Dc^	1017.60 ± 9.27^Cc^	↑ (15.43)	3993.10 ± 4.95^Ba^	4725.80 ± 11.97^Aa^	↑ (18.35)
**SC**	888.21 ± 7.57^Dc^	1012.69 ± 16.97^Cc^	↑ (14.01)	3166.40 ± 44.12^Ab^	1521.77 ± 19.19^Bc^	↓ (51.94)

* The percentage of decrease/increase in the content of phenolics compounds value from the EPC to the SPE is indicated as ↑↓ (%).

** (^a–d^) Means ± standard deviation in a row without common superscripts are significantly different at *p* < 0.05, Tukey test indicated by capital letter (^A–D^) is the effect of the method used while each column indicated by small letter is the effect of different samples. *N* = 3 for all variables. GS: Green Ramon Seeds, RS: Roasted Ramon Seeds, FR: Fruit of Ramon, SC: Seed coat layer of Ramon seeds. EPC: extractable phenolic compounds, SPE: solid phase extraction.

Results showed significant differences (*p* < 0.05) between the two methods. In most cases, the values increased following SPE relative to untreated controls, using both methods. This suggests that SPE was generally ineffective in removing polar interferences, with the exception of the seed coat (SC) in the Fast Blue method, where a 51.94% decrease in phenolic content was observed. The highest total phenolic content was observed in RS (2933.94 mg GAE/100 g) as measured by the Folin–Ciocalteu assay, compared to GS (2883.20 mg GAE/100 g). These findings are similar to those reported by Quintero-Hilario et al.,^3^ who reported higher phenolic content in medium and high roasted seeds (894.78 mg GAE/100 g and 1337.19 mg GAE/100 g, respectively) compared to fresh green seeds (271.58 mg GAE/100 g). The increased phenolic content in roasted seeds may result from the thermal release of bound phenolics and flavonoids due to the cell wall disruption, as well as from Maillard reaction products that can react with the Folin–Ciocalteu reagent, thus contributing to the increase in total phenolic compounds.^57^

This study analyzed various ANFs in the different parts of the Ramon seed. Figure 1 shows the variability in the antinutrient content across the seed components. Saponins, produced in a variety of plant species as secondary metabolites, are widely used in medical and pharmaceutical applications.^58^ However, they can interact with other food components (e.g., proteins, lipids, and minerals) to form complexes, thus reducing their bioavailability.^59^ In this study, the SC exhibited the highest saponin content (1337.58 mg DE/100 g), followed by the whole seeds (961.10 mg DE/100 g). Similar findings have been reported in quinoa, where raw seeds contained higher saponin levels than polished seeds due to the removal of the perianth during polishing, which otherwise serves as a protective layer during seed maturation and storage.^60^

**Figure 1:**
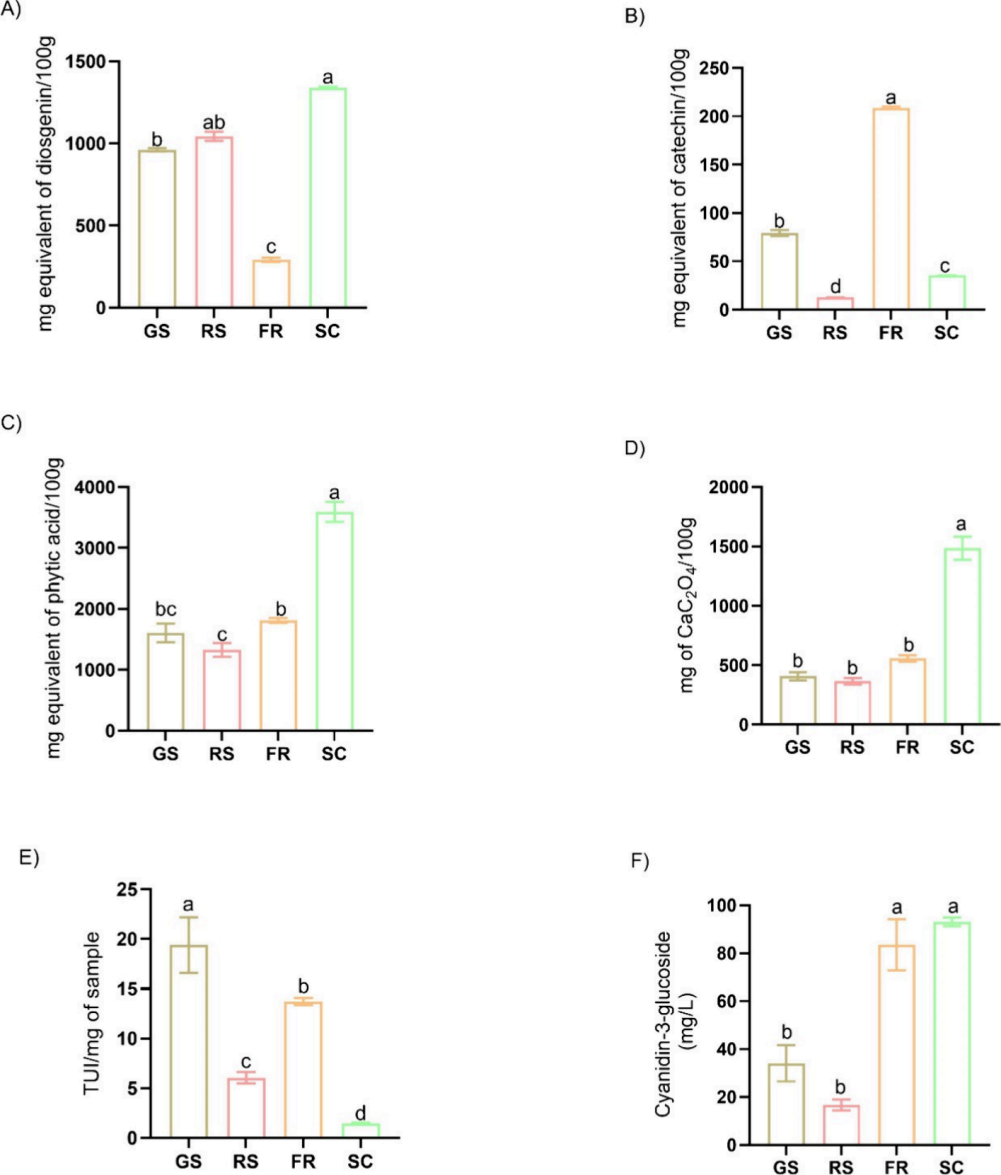
Antinutritional factors composition of green Ramon Seeds (GS), roasted Ramon seeds (RS), fruit of Ramon (FR), seed coat layer of Ramon seed (SC) flours. (A) Saponins, (B) Tannins, (C) Phytic acid, (D) Oxalates, (E) Trypsin inhibitor activity, and (F) Anthocyanins. CaC_2_O_4_: Calcium oxalate, TUI: Trypsin inhibitor units. Nonequal letters above bars indicate statistical differences by the Tukey test (*p* < 0.05).

Tannins, a class of polyphenolic compounds with known medicinal and therapeutic properties, function as antioxidants and exhibit diverse pharmacological activities, including anti-inflammatory and anticancer effects.^61^ In contrast, tannins, which impact astringency properties, are also considered as ANFs due to their ability to reduce nutrient digestibility and inhibit enzyme activity.^62,63^ In this study, the highest tannin content was observed in the fruit (208.66 mg of CE/100 g). Tannins were found to decrease in mature and ripening fruits. Within the seed fractions, GS had a higher tannin content (79.34 mg CE/100 g) compared to that of RS (12.67 mg CE/100 g). These results differ from those reported by Quintero-Hilario et al.,^3^ who reported an increase in tannin content after roasting. The discrepancy may be attributed to differences in the roasting conditions. The previous study employed roasting at 90 °C for 20–35 min, whereas the present study used a milder temperature of 60 °C for 72 h.

The phytic acid content in GS exceeds that reported for many legumes, such as raw fava bean (1224 mg/100 g), raw chickpea (1114 mg/100 g), and raw red lentil (1075 mg/100 g).^64^ Phytic acid content did not differ significantly between GS and RS, but it was slightly higher in the SC. Similar findings were reported by Osuna-Gallardo et al.,^65^ who found no effect in phytic acid content following cooking of ayocote-bean flour; this was mainly due to the relative heat stability of phytates. Additionally, Han et al.^66^ also reported only minor reductions in phytic acid content after extrusion of oilseed cakes blended with pea or hemp protein ingredients, supporting the notion that conventional thermal and mechanical treatments do not fully eliminate phytate content. This contrasts with findings by Chauhan et al.,^67^ who observed a significant reduction in phytic acid content in black soybean after roasting for 2 min, from 1.41 to 1.22% resulting in a 13.47% reduction. Phytic acid serves as a phosphorus storage compound and provides antioxidant protection to developing seeds.^68^ However, it can chelate essential mineral cations such as Cu^2+^, Ca^2+^, Zn^2+^, and Fe^3+^, thus reducing their absorption.^69^

Oxalates, which are involved in calcium regulation, defense, and detoxification in plants, also pose nutritional concerns in humans due to their ability to chelate minerals and contribute to kidney stone formation.^68,70^ The highest oxalate content was found in the SC (1431.48 mg CaC_2_O_4_), consistent with previous studies showing that CaOx crystals are concentrated in the sclerenchyma tissue of seed coats, such as in pecan nutshells.^71^ Identifying the location of antinutrient compounds is crucial, especially for improving protein extraction efficiency and techno-functional properties. There is no general legal maximum for oxalates in foods and no official daily intake limit set by major regulators (EFSA, Food Standards Agency, FDA). Clinical guidance exists only for people at risk of kidney stones. However, typical dietary exposure in healthy adults is commonly reported around ∼50–200 mg/day (highly variable by diet).^72^ For individuals with calcium-oxalate kidney stone risk, many clinical resources recommend a “low-oxalate” diet of ∼40–50 mg/day, alongside normal calcium intake to bind oxalate in the gut. Food processing, particularly boiling and soaking, have been shown to lower the soluble oxalate fraction, which is the more absorbable form.^73,74^

Trypsin inhibitors are predominantly present in most legume crops, such as soybeans and beans, and are known to reduce protein digestibility, inhibit nutrient absorption, and impair growth by inducing pancreatic hypertrophy.^34^ In plant-based foods, trypsin inhibition is mainly attributed to protease inhibitors, especially those of the Kunitz and Bowman–Birk families. These proteinaceous compounds interact with the active site of trypsin, blocking enzymatic activity and thus reducing protein hydrolysis.^75–77^ In this study, trypsin inhibitor activity was lower in RS (6.37 TIU/mg) compared with GS (17.78 TIU/mg), suggesting that thermal processing reduces their activity. These results are in agreement with those of Zhou et al.,^78^ who reported significant reductions in trypsin inhibitor activity in yellow and black beans after roasting at 210 °C (1696.5 TIU/g to 324.8 TIU/g, and 1375.8 TIU/g to 923.5 TIU/g, respectively).

Anthocyanins are water-soluble flavonoids responsible for the colored pigments in most vegetables, fruits, and plant species.^79^ In this study, the highest anthocyanin content was found in SC (93.09 mg C3GE/L), as shown in Figure 1. The higher concentration of anthocyanins in the seed coat layer may be due to their localization in the outer layer of the seeds. This pattern is similar to that observed in cereals such as wheat, where anthocyanins are predominantly localized in the pericarp and aleurone layers of the grain.^79^ Additionally, the dark color exhibited by SC may be attributable to its higher anthocyanin content, similar to those findings by Manzanilla-Valdez et al.,^80^ who reported that black quinoa contains higher anthocyanin levels compared to yellow and red varieties, owing to its pigment-rich seed coat. In the present study, processing led to a reduction of anthocyanin content; GS retained substantially more anthocyanins than RS. These findings are in line with those of Osuna-Gallardo et al.,^65^ who found that cooking decreased anthocyanin content, with retention of approximately 61%, compared to the raw ayocote bean flour.

Lectins are carbohydrate-binding glycoproteins capable of agglutinating cells or precipitating glyco-conjugates.^81,82^ In this study, lectin activity was evident in GS and FR (at high concentrations), as indicated by the formation of a diffuse red suspension in microplate assays (Figure 2). Lectins were not detectable at a concentration starting from 0.37 mg/mL for GS and 0.33 mg/mL for FR, which differs from Manzanilla-Valdez et al.^80^ study, where no lectins were detected in the range of concentration between 0.94 to 1.39 mg/mL for yellow, red, and black quinoa varieties. However, lectin activity diminished at lower concentrations and was absent in RS, suggesting that roasting deactivates these proteins. A study by González-Cruz et al.^83^ found that the hemagglutination activity of ayocote beans is lost at temperatures above 90 °C, likely due to heat-induced alterations in the protein secondary structure of lectins.^84^ Additionally, Osuna-Gallardo et al.^65^ reported the same findings, where cooked ayocote bean flour showed no activity at all concentrations tested, suggesting that the cooking process was the most efficient in suppressing hemagglutinin activity.

**Figure 2:**
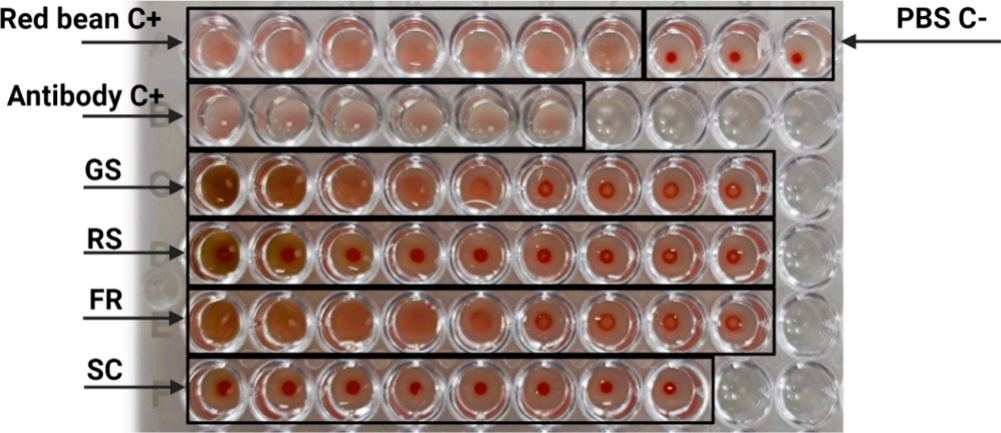
Hemagglutination activity of green Ramon seeds (GS), roasted Ramon seeds (RS), fruit of Ramon (FR), seed coat layer of Ramon seeds (SC) flour. Red bean lectin (C+): 0.8, 0.4, 0.2, 0.1, 0.05, and 0.025 mg/mL. Positive control (C+) Antibody: 0.8, 0.4, 0.2, 0.1, 0.05, and 0.025 mg/mL. Negative control (C−) PBS: 25 mL per well. GS: 5.9, 2.95, 1.48, 0.74, 0.37, 0.18, 0.09, 0.046, and 0.023 mg/mL, RS: 3.75, 1.88, 0.94, 0.47, 0.23, 0.12, 0.06, 0.03, and 0.01 mg/mL, FR: 5.26, 2.63, 1.31, 0.66, 0.33, 0.16, 0.08, 0.04, and 0.02 mg/mL, SC: 0.54, 0.27, 0.13, 0.07, 0.03, 0.02, 0.008, and 0.004 mg/mL.

### 3.4 Protein Characterization

#### 3.4.1 Sodium Dodecyl Sulfate-Polyacrylamide Gel Electrophoresis (SDS-PAGE) Analysis

The SDS-PAGE profile for Ramon seed flour is presented in Figure 3. To date, there are limited publications detailing the molecular weight distribution of proteins in Ramon seed flour, particularly with regard to its fruit and seed coat layer fractions. In this study, the most prominent bands were observed at approximately 21 and 14 kDa under both reducing and non-reducing conditions. GS showed distinct protein bands at 21.6 and 14 kDa, along with faint bands at 9–12 kDa under reducing conditions. A similar pattern was observed in RS samples, though with lower band intensity compared to GS. In RS, bands appeared at approximately 21.6 kDa, followed by 14.5 kDa and a range of 9–12 kDa. In contrast, the protein profiles of FR and SC samples revealed bands beginning around 14 kDa under both non-reducing and reducing conditions. Notably, the SC samples exhibited additional low-intensity bands at molecular weights ranging from 2–6 kDa. These results suggest that the raw Ramon seed flours are characterized by a predominance of low molecular weight proteins, with major bands appearing below 25 kDa and particularly concentrated around 14–21 kDa.

**Figure 3:**
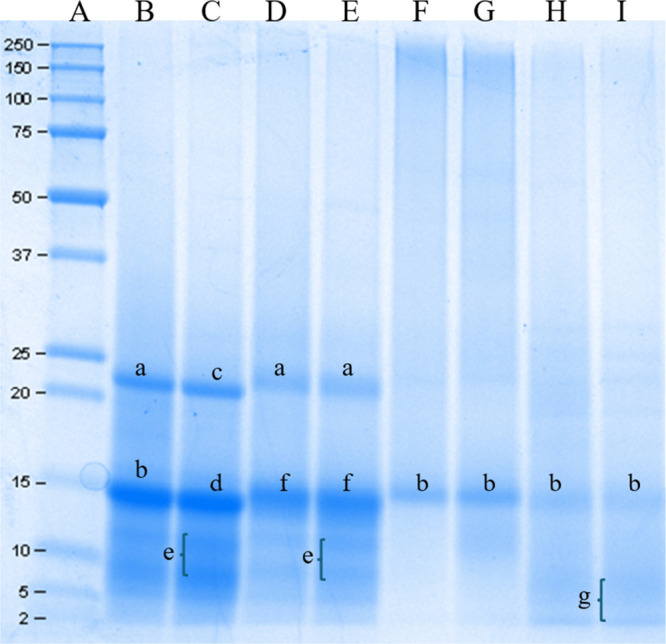
Protein electrophoretic profile of green Ramon seeds (GS), roasted Ramon seeds (RS), fruit of Ramon (FR), and seed coat layer of Ramon seeds (SC) flours under reducing and non-reducing conditions. A: standard (kDa), B: green Ramon seeds (GS) in non-reducing conditions, C: green Ramon seeds (GS) under reducing conditions, D: roasted Ramon seeds (RS) under non-reducing conditions, E: roasted Ramon seeds (RS) under reducing conditions, F: fruit of Ramon (FR) under non-reducing conditions, G: fruit of Ramon (FR) under reducing conditions, H: seed coat layer of Ramon seeds (SC) under non-reducing conditions, and I: seed coat layer of Ramon seeds (SC) under reducing conditions. Protein bands, a: 21.6 kDa, b: 14.0 kDa, c: 21.1 kDa, d: 13.8 kDa, e: 9–12 kDa, f: 14.5 kDa, and g: 2–6 kDa.

#### 3.4.2 Proteomics Analysis of Ramon Seed Flour

Proteomics analysis was conducted based on SDS-PAGE results, where four prominent bands ranging from approximately 21 to 8 kDa were selected for protein identification in Ramon seeds. The proteins present in these bands were analyzed via mass spectrometry, and the results are summarized in Table 4, highlighting the most abundant proteins identified in Ramón seed samples. Band 1 was primarily composed of β-amylase (fragment), an enzyme involved in starch degradation through the hydrolysis of α,1–4 glycosidic linkages in starch and related polysaccharides.^85^ Band 2 shown was enriched with α-1,4-glucan phosphorylase (fragment), an enzyme that catalyzes the reversible phosphorolysis of α-1,4-linked polysaccharides such as glycogen, starch, and maltodextrins. This process is essential for the mobilization and utilization of storage carbohydrates.^86^ Glyceraldehyde-3-phosphate dehydrogenase (fragment) was predominantly identified in Band 3. This enzyme catalyzes the conversion of glyceraldehyde-3-phosphate to 1,3-bisphosphoglycerate with the concurrent reduction of NAD^+^ to NADH, a key step in the glycolytic pathway. This reaction plays a critical role in energy production and provides precursors for various anabolic processes.^87^ In band 4, catalase (fragment) was identified as the most abundant protein. Catalase is a key antioxidant enzyme that catalyzes the decomposition of hydrogen peroxide into oxygen and water via a two-step reaction, thereby protecting cells from oxidative damage.^88^

**Table 4:** Proteomics Analysis of Ramon Seeds Flour Proteins Obtained from Mass Spectrometry Analysis[Table-fn t4fn1]

Bands letters	Description	Peptides	Unique peptides	MW (kDa)	Accession
a,c	β-amylase (fragment)	5	5	5869	W9S2P6; A0A0A0R213
b,d,f	α-1,4 glucan phosphorylase (fragment)	11	9	1131	W9QTH0; W9SE54
e	glyceraldehyde-3-phosphate dehydrogenase (fragment)	6	2	3403	W9S8K2
g	catalase (fragment)	12	8	5700	A0A6G8IRL7; W9RJ43

a-g Band letters are referred from Figure 3.

### 3.5 Amino Acid Profile and Protein Quality Assessment

The amino acid profile of Ramon flour is listed in Table 5. Asp and Asn were the most abundant amino acids found across all samples, accounting for more than >8.14% of the total amino acid content. EAA represented between 40.13–43.61% of the total amino acids, with the highest proportion observed in the seed coat layer (43.61%) and the lowest in roasted seeds (40.13%). Among the EAAs, Leu was the most abundant in all samples, ranging from 6.57–8.13 g/100 g of protein, thus meeting the daily requirements for children as outlined by FAO/WHO (1985).^89^ Among the nine indispensable amino acids, Val, Ile, and Trp levels remained relatively consistent before and after roasting. These results differ slightly from those of a previous study, which reported Phe, Thr, and His as the most stable during roasting.^9^ This discrepancy may be attributed to differences in processing temperature, as the previous study used a higher roasting temperature (160 °C) compared to the current study. Notably, Lys was the most affected by roasting, showing a decrease of 27%, likely due to the high temperature resulted in a decrease in Lys content, directly linked to its role as a reactive amine in the Maillard reaction under heat treatment.^90^

**Table 5:** Amino Acid Profile of Green Ramon Seeds (GS), Roasted Ramon Seeds (RS), Fruit of Ramon (FR), and Seed Coat Layer of Ramon Seeds (SC) Flours (g/100 g of Protein)[Table-fn t5fn1]

					FAO/WHO (1985)	
Amino acid	GS	RS	FR	SC	Children	Adults
Asp + Asn	23.30 ± 0.49^a^	23.67 ± 0.64^a^	17.00 ± 0.42^b^	18.67 ± 0.22^b^		
Glu + Gln	9.36 ± 0.07^b^	9.47 ± 0.50^b^	10.30 ± 0.23^b^	12.18 ± 0.02^a^		
Ser	5.19 ± 0.04^b^	5.22 ± 0.10^b^	7.11 ± 0.04^a^	5.59 ± 0.05^c^		
His	2.05 ± 0.07^a^	1.60 ± 0.06^c^	1.82 ± 0.03^b^	1.95 ± 0.02 ^ab^		
Gly	5.40 ± 0.02^b^	5.77 ± 0.11^c^	8.47 ± 0.04^a^	6.34 ± 0.05^d^		
**Thr**	**5.11 ± 0.02** ^**b**^	**5.00 ± 0.11** ^**b**^	**5.66 ± 0.14** ^**a**^	**5.68 ± 0.01** ^**a**^	**3.4**	**0.9**
Arg	6.86 ± 0.11^a^	5.59 ± 0.06^b^	5.08 ± 0.15^c^	5.02 ± 0.05^c^		
Ala	6.77 ± 0.04^b^	8.41 ± 0.16^a^	6.02 ± 0.06^c^	6.03 ± 0.18^c^		
Pro	6.42 ± 0.27^ab^	7.23 ± 0.37^a^	5.44 ± 0.38 ^b^	5.24 ± 0.21^b^		
**Val**	**4.26 ± 0.12** ^**a**^	**4.52 ± 0.13** ^**a**^	**5.35 ± 0.57** ^**a**^	**5.05 ± 0.13** ^**a**^	**3.5**	**1.3**
**Met + Cys**	**1.21 ± 0.05** ^**b**^	**0.93 ± 0.04** ^**d**^	**1.75 ± 0.01** ^**a**^	**1.13 ± 0.01** ^**c**^	**2.5** [Table-fn t5fn2]	**1.7** [Table-fn t5fn2]
**Ile**	**4.06 ± 0.01** ^**b**^	**4.30 ± 0.05** ^**c**^	**4.63 ± 0.01** ^**d**^	**4.98 ± 0.01** ^**a**^	**2.8**	**1.3**
**Trp**	**0.75 ± 0.01** ^**a**^	**0.79 ± 0.01** ^**ab**^	**0.74 ± 0.02** ^**a**^	**0.75 ± 0.03** ^**ab**^	**0.8**	**0.5**
**Leu**	**6.58 ± 0.02** ^**b**^	**6.57 ± 0.09** ^**b**^	**7.91 ± 0.06** ^**a**^	**8.13 ± 0.01** ^**a**^	**6.6**	**1.9**
**Phe + Tyr**	**7.63 ± 0.02** ^**bc**^	**7.24 ± 0.47** ^**c**^	**8.22 ± 0.54** ^**a**^	**7.87 ± 0.19** ^**ab**^	**6.3** [Table-fn t5fn3]	**1.9** [Table-fn t5fn3]
**Lys**	**5.05 ± 0.01** ^**b**^	**3.69 ± 0.08** ^**c**^	**4.50 ± 0.03** ^**d**^	**5.39 ± 0.01** ^**a**^	**5.8**	**1.6**

* (a−d) Different lowercase letters within each row indicate significant differences (*p*-value <0.05), Tukey test (*p* < 0.05). *N* = 3 for all variables. FAO/WHO (1985) pattern was used as a reference. GS: Green Ramon Seeds, RS: Roasted Ramon Seeds, FR: Fruit of Ramon, SC: Seed coat layer of Ramon seeds. Gly: glycine, Lys: lysine, Gln: glutamine, Glu: glutamic acid, Ser: serine, Ala: alanine, Leu: leucine, Met: methionine, Phe: phenylalanine, Trp: tryptophan, Pro: proline, Val: valine, Ile: isoleucine, Cys: cysteine, Tyr: tyrosine, His: histidine, Arg: arginine, Asn: asparagine, Asp: aspartic acid, Thr: threonine. Letters in bold are essential amino acids.

A Met + Cys.

B Phe + Tyr.

The EAAI values shown in Table 6 varied among the samples, with the highest values observed in FR (131.46%), followed by SC (140.74%). Generally, an EAAI value above 90% is considered indicative of high nutritional quality, while values between 80–90% are considered useful, and values below 70% are regarded as inadequate. Additionally, the biological value (BV), which reflects the proportion of absorbed protein that becomes incorporated into the body’s proteins, ranged from 70–90% in FR and SC - considerably higher than that of whole seeds.^91,92^ These values also exceed those of oat flour, which has reported EAAI and BV values of 44.55% and 36.86%, respectively.^93^ EAAI values are comparable to those reported for maize (*Zea mays*) and common bean (*Phaseolus vulgaris*) (EAAI = 61.8–151.4%), and notably higher than those of pumpkin seeds (*Cucurbita* spp.) (EAAI = 2.2–46.4%).^94^ These species are primary protein sources for populations in the Yucatán Peninsula, Mexico, where Ramón seeds are traditionally consumed. Regarding biological value (BV), the results obtained in this study are similar to those reported for legumes, such as flours of raw black and navy beans (*Phaseolus vulgaris*) (BV = 92.3–106.9), and superior to those of raw red pea (*Pisum sativum*), chickpea (*Cicer arietinum*), and fava bean (*Vicia faba*) flours (BV = 35.2–67.6).^95^ AAS was higher in GS (0.48) than in RS (0.37), suggesting that processing may negatively influence the amino acid quality of the samples. These AAS values are comparable to those reported for pumpkin seeds,^94^ a traditional snack in the regions where the Ramón tree grows, indicating that Ramón could also serve as a potential alternative snack for local populations. The PER, which assesses the effectiveness of a protein in promoting growth by measuring weight gain per gram of protein consumed, was above 2.0 for most samples. The highest PER value derived from theoretical PER calculations was found in SC, suggesting good protein quality.^96^ These findings align with previous reports for maize and common bean flours, particularly their PER_1–2_ values (PER_1–2_ > 2.0).^94^ Theoretical and *in vivo* protein-efficiency ratio (PER) values can differ. The *in vivo* PER directly measures the effect of a protein on growth in rats,^40^ ,^80^ whereas theoretical PER is calculated from equations that account for the content of 12 amino acids; although the theoretical approach is more conservative, it provides a useful estimate of protein efficiency.^40,41,80^ In the context of these protein quality parameters, Ramon fruit/seed ingredients could represent an alternative source to maize, beans, and pumpkin seeds, the key plant proteins obtained from the ancestral Maya milpa polyculture system.

**Table 6:** Protein Quality Parameters of Green Ramon Seeds (GS), Roasted Ramon Seeds (RS), Fruit of Ramon (FR), and Seed Coat Layer of Ramon Seeds (SC) Flours^a^

Sample	IVPD (%)	AAS	EAAI (%)	BV (%)	PER_1_	PER_2_	PER_3_	PER_4_	PER_5_	IVPDCAAS (%)
GS	68.10 ± 1.00^b^	0.48(SAA)	99.92	97.21	2.01	2.26	1.59	2.37	2.50	32.96
RS	67.34 ± 0.29^b^	0.37(SAA)	92.12	88.71	1.97	2.28	1.67	2.27	2.31	25.05
FR	70.73 ± 1.10^a^	0.67(Trp)	131.46	131.59	2.67	2.81	2.20	2.64	2.62	47.58
SC	68.28 ± 0.38^b^	0.45(SAA)	140.74	141.71	2.77	2.99	3.22	2.78	2.68	30.86

a GS: Green Ramon Seeds, RS: Roasted Ramon Seeds, FR: Fruit of Ramon, SC: Seed coat layer of Ramon Seeds, SAA: sulfur amino acids (Met+Cys), Trp: Tryptophan. Different lowercase letters within each column indicate significant differences (p-value <0.05), Tukey test (*p* < 0.05). Note: EAAI%, AAS%, BV, PER_1_, PER_2_, PER_3_, PER_4_, PER_5_ and IVPDCAAS are calculated values, no standard deviation is available. IVPD: *in vitro* protein digestibility, AAS: amino acid score, EAAI: essential amino acid index, BV: biological value, PER: protein efficiency ratio, IVPDCAAS: *in vitro* protein-digestibility corrected amino acid score.

The *in vitro* protein digestibility (IVPD) was higher in FR; however, no significant difference (*p* > 0.05) was found between GS (68.10%) and RS (67.34%). This suggests that these seeds may benefit from alternative processing methods to further enhance protein digestibility. Similar findings were also found for fava beans, where no significant differences (*p* > 0.05) in IVPD were found between extruded (82.22%), cooked (81.41%), and baked (76.79%) samples.^42^ IVPDCAAS ranged from 25.05–47.58%. These values were slightly higher than those reported for quinoa flour, which ranges from 23.96–34.92%.^25^ However, the IVPDCAAS values in this study are considered relatively low, likely due to limited protein digestibility. One contributing factor may be the presence of antinutritional compounds and the high content of dietary fiber, as previously discussed. For instance, the lower IVPDCAAS observed in RS may be attributed to its higher saponin content, which could facilitate the formation of saponin–protein complexes that impair digestibility. These findings highlight the nutritional potential of Ramón seeds, particularly in terms of protein quality and digestibility, which are comparable to cereals (wheat, rice, corn).^97^

Protein quality indexes provide complementary perspectives on nutritional value. EAAI offers a rapid evaluation based on amino acid composition but does not account for digestibility. PER, traditionally derived from animal growth studies, can be predicted from amino acid profiles but may not fully represent human requirements. IVPDCAAS combines digestibility and amino acid composition, offering a more physiologically relevant estimate for human nutrition. However, it can be influenced by the reference pattern used and the limitations inherent to *in vitro* digestibility assays. Using multiple protein quality indexes together allows for a more comprehensive and robust assessment of protein quality, while recognizing the specific constraints of each method.^98^

### 3.6 Gluten Analysis

The presence of gluten was previously analyzed, and it was concluded that Ramon seed flour is free from gluten (data not shown).^99^ Gluten proteins are the primary storage proteins found in cereal grains, where they play a crucial role in facilitating germination and seedling growth. These proteins are predominantly localized within the starchy endosperm and are not present in other grain or plant tissues.^100^ In the present study, Ramón flour tested negative for gluten, further supporting earlier findings that these plant fractions are naturally gluten-free. A previous study by Carter and Northcutt^9^ also reported negative results for raw*Brosimum alicastrum* flour, using a sensitivity threshold of 10 ppm. Gluten is considered essential in most baked goods due to its unique ability to form a cohesive dough structure and contribute to the viscoelastic properties required for products such as bread and pasta.^100^ However, gluten proteins are also known to trigger adverse health reactions in individuals with celiac disease or gluten sensitivity. Symptoms may include diarrhea, fatigue, weight loss, bloating, and anemia. For this reason, strict adherence to a gluten-free diet is critical for affected individuals to prevent the onset of symptoms.^101^

This study investigated the nutritional and functional potential of different components of Ramón seeds, including seed coat (SC), fruit (FR), and both roasted (RS) and green (GS, unprocessed) seeds. The findings highlighted that Ramon seeds are a promising and sustainable source of nutrients, owing to their substantial protein and carbohydrate content, as well as a favorable amino acid profile. Notably, the seeds—particularly when roasted—exhibited high levels of bioactive phenolic compounds, demonstrating significant antioxidant activity even after processing.

However, the presence of ANFs such as saponins, phytic acid, oxalates, and tannins was also identified, which may impair nutrient digestibility and bioavailability. This underscores the importance of selecting appropriate processing methods to mitigate these effects. Despite this limitation, the results suggest that Ramón seeds, with tailored processing methods, could serve as a valuable plant-based protein source comparable to conventional cereal grains. Their gluten-free nature and favorable protein quality indicators, such as the essential amino acid index (EAAI) and low to moderate protein digestibility, further support their potential for inclusion in functional and specialized food products. Further research is needed to elucidate the full impact of antinutritional compounds on human health and optimize processing strategies for industrial-scale applications. Overall, Ramón seeds offer a promising opportunity to enhance food security and support the development of sustainable, nutrient-dense diets.

## Supplementary Material



## Notes

Overview diagram of different components of Ramon seed including the seed coat, fruit and both roasted and green (unprocessed) seeds (PDF)

## Abbreviations

AAS: amino acid score
ANFs: antinutritional factors
BV: biological value
EAA: essential amino acids
EAAI: essential amino acid index
EPP: extractable phenolic compound
FBBB: fast blue BB
FR: fruit of Ramon
GS: green seeds
IVPD: in vitro protein digestibility
IVPDCAAS: in vitro protein digestibility corrected amino acid score
LMW: low molecular weight
PER: protein efficiency ratio
RS: roasted seeds
SPE: solid-phase extraction
TIU: trypsin inhibitory unit
