# Supplementary material for: Nutritional Value, Antinutritional Factors, and Protein Quality of Brosimum alicastrum Seeds: A Sustainable Protein Source
Source: J Agric Food Chem. 2025 Sep 5;73(37):23580–94. doi: 10.1021/acs.jafc.5c06101 (PMC12447510; doi:10.1021/acs.jafc.5c06101)
Supplement: Supplementary file 1 [file jf5c06101_si_001.pdf]

Supporting Information

Nutritional Value, Antinutritional Factors, and Protein Quality of *Brosimum alicastrum* Seeds: A Sustainable Protein Source

Hani Farhana Nazir[a], Raul Tapia-Tussell[b*], Matthew G. Nosworthy[c,d], Oscar Abel Sánchez-Velázquez[a], Adam Dowle[e], Idolo Ifie[a], Alan Javier Hernández-Álvarez[a,f*]

[a]School of Food Science & Nutrition, University of Leeds, LS2 9JT, Leeds, UK

[b]Renewable Energy Unit, Yucatan Scientific Research Center, Sierra Papacal, Merida 97302, Mexico

[c]Guelph Research and Development Centre, Agriculture and Agri-Food Canada, Guelph, Ontario, Canada, N1G 5C9
[d]College of Pharmacy and Nutrition, University of Saskatchewan, Saskatoon, Saskatchewan, Canada, S7N 5E5
[e]Bioscience Technology Facility, Department of Biology, University of York, York YO10 5DD, UK
[f]National Alternative Protein Innovation Centre, NAPIC, UK

*Corresponding authors:
a.j.hernandezalvarez@leeds.ac.uk (Alan Javier Hernández-Álvarez)
rtapia@cicy.mx (Raul Tapia-Tussell)

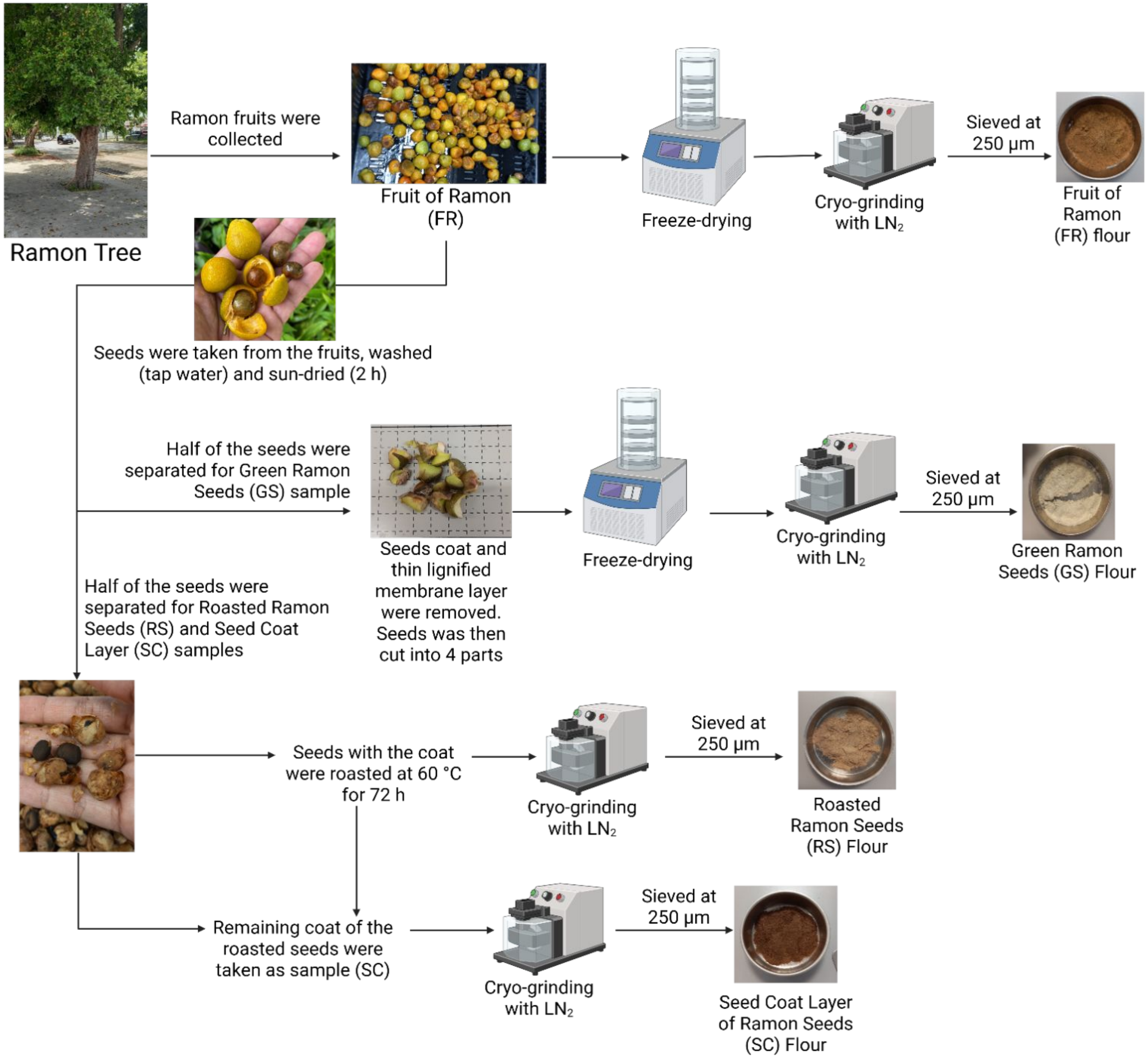

Figure S1. Overview diagram of different components of Ramon seed including the seed coat, fruit and both roasted and green (unprocessed) seeds.
